# The Role of Binocular Disparity in Stereoscopic Images of Objects in the Macaque Anterior Intraparietal Area

**DOI:** 10.1371/journal.pone.0055340

**Published:** 2013-02-07

**Authors:** Maria C. Romero, Ilse C. L. Van Dromme, Peter Janssen

**Affiliations:** Laboratorium voor Neuro- en Psychofysiologie, KULeuven, Leuven, Belgium; University College London, United Kingdom

## Abstract

Neurons in the macaque Anterior Intraparietal area (AIP) encode depth structure in random-dot stimuli defined by gradients of binocular disparity, but the importance of binocular disparity in real-world objects for AIP neurons is unknown. We investigated the effect of binocular disparity on the responses of AIP neurons to images of real-world objects during passive fixation. We presented stereoscopic images of natural and man-made objects in which the disparity information was congruent or incongruent with disparity gradients present in the real-world objects, and images of the same objects where such gradients were absent. Although more than half of the AIP neurons were significantly affected by binocular disparity, the great majority of AIP neurons remained image selective even in the absence of binocular disparity. AIP neurons tended to prefer stimuli in which the depth information derived from binocular disparity was congruent with the depth information signaled by monocular depth cues, indicating that these monocular depth cues have an influence upon AIP neurons. Finally, in contrast to neurons in the inferior temporal cortex, AIP neurons do not represent images of objects in terms of categories such as animate-inanimate, but utilize representations based upon simple shape features including aspect ratio.

## Introduction

The analysis of visual information about objects takes place in the primate brain both in the ventral and in the dorsal visual stream [1], [2]. The Anterior Intraparietal area (AIP) is a key end-stage area of the dorsal stream that is crucial for object grasping [3]. Many AIP neurons respond to real-world objects [4]–[6] during object grasping and during passive fixation. Furthermore, AIP neurons encode the three-dimensional (3D) structure of shapes defined by binocular disparity [7], [8]. Recently, we also showed that a subpopulation of AIP neurons may encode two-dimensional shape features across a limited number of spatial positions and sizes [9].

Stereopsis is important for visually-guided grasping [10], but no study has investigated the influence of binocular disparity on the responses of AIP neurons to images of real-world objects. In the studies that have tested AIP neurons with random-dot stereograms [7], [8], [11], [12] binocular disparity was the only available depth cue, and neural selectivity for concave versus convex surfaces was assessed. However real-world objects furnish depth information from a variety of sources in addition to disparity, and the influence of binocular disparity in images of objects containing monocular depth information upon AIP neurons has never been investigated. Therefore, the first objective of this study was to assess the influence of disparity information in images of real-world objects, on AIP neurons. Secondly, it is not clear how AIP neurons encode object images. We have previously shown that for the great majority of AIP neurons, the silhouette or outline of a shape is sufficient to evoke selective responses [9], but it is unclear how these stimuli are represented within the neural space of AIP. For example, in the Inferior Temporal Cortex (ITC) the categorical structure of objects, most notably the animate-inanimate category, is represented in the responses of a population of neurons [13]. Thus we also wanted to determine whether the intuitive category structure across a set of object images is represented in the combined responses of a population of AIP neurons.

We found that although binocular disparity exerted a significant influence on the responses of half of the AIP neurons recorded, the great majority of these neurons still showed significant image selectivities in the absence of disparity. Monocular depth cues had a weak but consistent effect on the disparity selectivity of AIP neurons, since most AIP neurons preferred stimuli in which the disparity information was congruent with the depth information from monocular cues. Furthermore, in contrast to the ITC, the responses in our population of AIP neurons did not reveal intuitive object categories, but appeared to reflect simple shape features such as elongation, in agreement with recent psychophysical evidence [14].

## Materials and Methods

### Surgical and Recording Procedures

The experimental protocol was similar to that previously described elsewhere [7], [9], [15]. All experimental procedures were performed in accordance with the National Institutes of Health Guide for the Care and Use of Laboratory Animals and EU Directive 2010/63/EU, and approved by the Ethical Committee at the Katholieke Universiteit Leuven. The animals in this study were pair-housed with cage enrichment (toys, foraging devices) at the primate facility of the KU Leuven Medical School. They were daily fed with standard primate chow supplemented with nuts, raisins, prunes and fruits if necessary. The monkeys received their daily water supply during the experiments, and we measured their weight daily. We reconstructed the recording positions using in vivo anatomical MRI. Monkey H is currently engaged in experiments, monkey M was sent to a primate sanctuary as part of the primate retirement program of KU Leuven.

One male and one female rhesus monkeys (monkey H, 6.5 kg; monkey M, 6 kg) were trained to sit in a primate chair. A head post (Crist Instrument for monkey H, custom-made for monkey M) was implanted on the skull using ceramic screws and dental acrylic. For this and all other surgical procedures, monkeys were kept under isoflurane anesthesia (1%) and strict aseptic conditions were maintained. Intensive training in passive fixation started after six weeks of recovery. Once animals acquired an acceptable level of performance, a craniotomy guided by anatomical MRI (Horsley-Clark coordinates 2P, 12L) was made and a recording cylinder implanted, oriented vertically above the IPS. A second cylinder was also attached in the coronal plane of the left and right hemispheres of monkey H, and on the right hemisphere of monkey M. This cylinder, located orthogonal to the one for recordings, allowed the real-time imaging of the electrode tip in the lateral bank of the IPS by means of a high-resolution ultrasound (Philips HDI 5000 SonoCT; scan frequency: 17 MHz; resolution: 0.5 mm). In order to verify the recording positions, glass capillaries were filled with a 2% copper sulfate solution and inserted into a recording grid at several positions during structural MRI (0.6 mm slice thickness). All recording instruments (cylinders and grid) were commercially available (Crist Instrument).

### Recording Procedures

During the experiment, we recorded single-unit activity at several grid positions using standard tungsten microelectrodes (impedance 1 MΩ at 1 kHz; FHC) inserted vertically through the dura by means of a 23 gauge stainless-steel guide tube and a hydraulic microdrive (FHC). The neural activity was amplified and filtered between 300 and 5000 Hz. Spike discrimination was carried out on-line by using a dual time window discriminator and displayed with LabView and custom-written software. The positions of both eyes were monitored using a binocular infrared-sensitive camera system (Eye Link II; SR Research) sampling the pupil position at 500 Hz. The stimuli were presented on a monitor (Vision Research Graphics M21L-67S01) equipped with a fast-decay P46-phosphor (VRG) and operating at 120 Hz. A photocell was attached to the stimulus display in the lower right corner, detecting the onset of a white square in the first video frame containing the stimulus. With this system, task-related timings could be precisely determined. All recorded signals (eye position, neural activity and photocell pulses) were digitized and processed at 20 kHz using a digital signal processor (DSP; C6000 series; Texas Instruments).

We searched for visually- responsive neurons during the performance of a passive fixation task. Especially during the initial recording sessions, the ultrasound cylinder would be filled with sterile conductive gel (Pharmaceutical Innovations) to visualize the electrode within various structures and to better determine the appropriate depth for recordings in the lateral bank of the IPS. The active-silent transitions observed between the white matter, medial bank, sulcus, and finally, the lateral bank of the IPS were useful for identifying the recording area.

### Stimuli and Tests

In the passive fixation task, each trial started with the presentation of a small square in the center of the screen (fixation point; 0.2 deg×0.2 deg). If both eyes remained within a 1 deg electronically-defined window around the fixation point for at least 500 ms, a visual stimulus was presented in the center of the display for the next 800 ms. Trials were considered correct when the animal held fixation until the offset of the stimulus, whereupon it received a drop of water as a reward.

**Figure 1:**
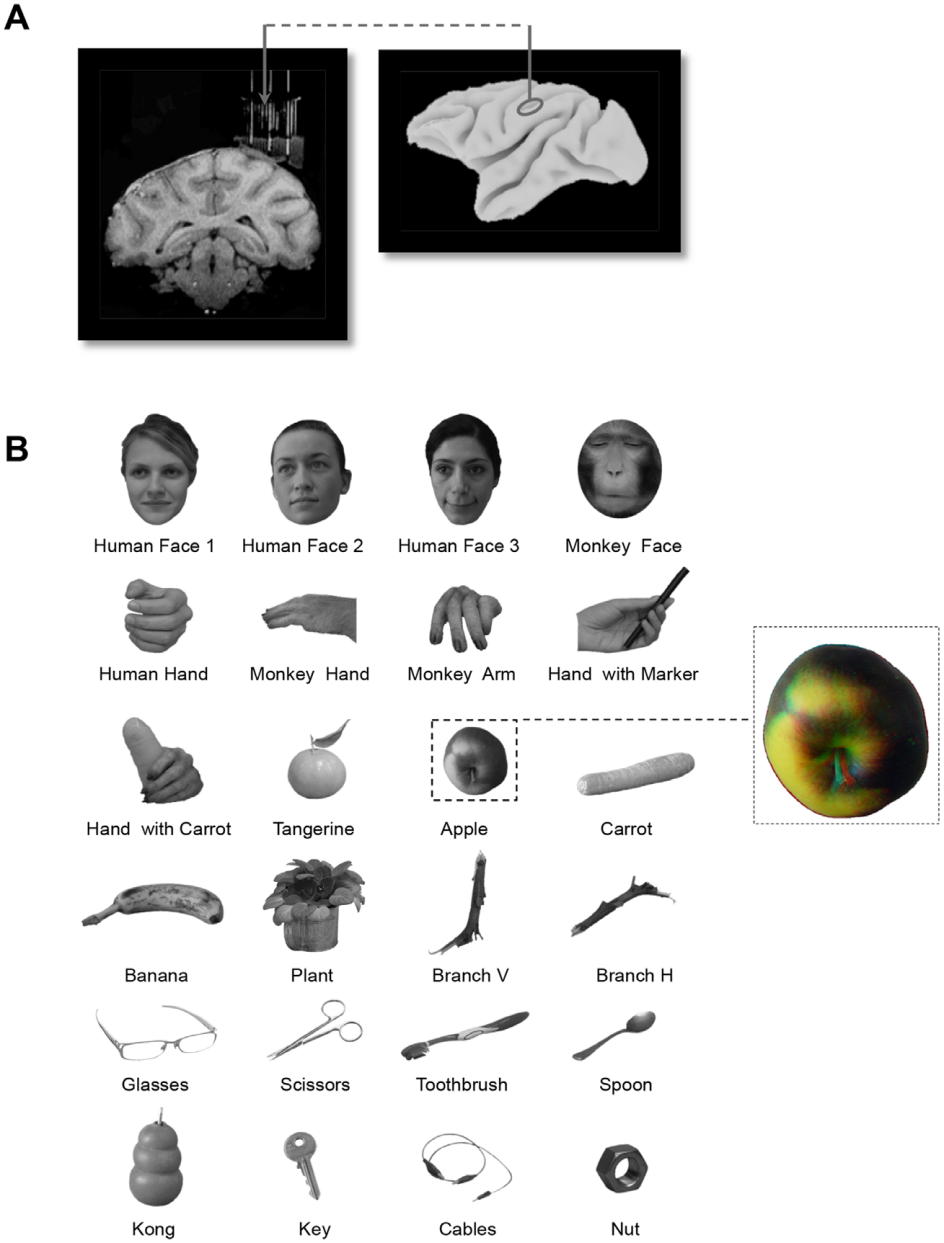
Anatomy and stimulus set. (A). Anatomical magnetic resonance image (MRI) and lateral view of the macaque brain, indicating the reconstructed recording positions in the AIP. (B). Monocular images are illustrated for all 24 images used in the Search Test. A red-green anaglyph version of the apple is shown in the inset (red in front of the left eye = congruent stereo mode).

Our basic stimulus set consisted of 24 stereoscopic images of natural and man-made objects, including faces (the individuals in the picture have given written informed consent -as outlined in PLOS consent form- to publish these case details), hands, fruits, branches and several artificial objects (Figure 1B). For each stereoscopic stimulus, we obtained the corresponding left and right eye images by taking two photographs from slightly different viewpoints matched to the interocular distance of the monkeys. The stimuli were presented stereoscopically by alternating the left and right eye images on the display, in combination with two ferroelectric liquid crystal shutters, each operating at 60 Hz, placed before monkeys’ eyes and synchronized to the vertical retrace of the monitor (two superimposed shutters for each eye; Displaytech). The use of a fast-decay phosphor and the two superimposed shutters prevented any measurable crosstalk between the eyes: no luminance could be detected behind the closed shutters during the presentations of the left or right images. For every stimulus in the basic set, the pattern of binocular disparity was congruent with the disparity gradients present in the real-world objects (congruent stereo set). We created a second set of stimuli composed of the same monocular images but containing the opposite disparity patterns by exchanging the left and right images (incongruent stereo set). Thus the congruent and incongruent stereo stimuli were composed of the same two monocular images. Finally, a third set of stimuli consisted of binocularly presented two-dimensional (2D) renderings of the same objects: for this stimulus set, the left and right eye shutters opened and closed simultaneously, synchronized to the appearance of either the left or right eye images on the display (no-stereo set). During the experiment, we also presented the stereoscopic stimuli monocularly by leaving one shutter closed while the other operated in synchrony with the appearance of the left or right eye images on the display (monocular mode). Stimuli differed in size (range of vertical and horizontal diameter: 1–9.8 deg) and surface area (range 6.9–109.3 deg^2^), but each object image (congruent, incongruent and no-stereo stimulus) contained the same monocular depth information, allowing a direct comparison of the neural responses to the different stereo modes. During the experiment, all stimuli were presented randomly interleaved on a black background at the fixation point, considering a viewing distance of 86 cm.

We searched for responsive AIP neurons during the presentation of congruent stereo stimuli (N = 48 neurons) or by intermingling both congruent and incongruent stereo stimuli (N = 91 neurons) at the fixation point (Search Test). Nonresponsive neurons were not studied further. If the neuron was responsive to at least one of these stimuli, we selected the object image evoking the strongest response (termed the *preferred* image) plus a second object image to which the neuron responded weakly (termed the *nonpreferred* image) for subsequent testing of the effect of disparity (Disparity Test). To this end, the preferred and nonpreferred images were presented in congruent stereo mode, incongruent stereo mode and no-stereo mode (binocularly), together with their monocular presentations (showed to the left and the right eyes independently). A subset of neurons showing significant response differences between the congruent and incongruent stereo modes of the same image was tested in a Position-in-Depth Test [7], in which the congruent and incongruent stereo stimuli of the preferred images were presented at five different positions in depth, ranging from -1 deg (near) to +1 deg (far).

### Data Analysis

All data analyses were performed using Matlab (Mathworks) except where noted differently. For each trial, the baseline firing rate was calculated from the mean activity recorded in the 400 ms interval preceding the stimulus onset. Net neural responses were then calculated by subtracting the baseline from the mean activity observed between 50 and 450 ms after the onset. For the Search Test, we calculated an S_width_ index [16], defined as (N-SUM/MAX)/(N-1), where N is the number of conditions, SUM is the sum of the responses, and MAX is the maximum response of the neuron. The S_width_ index was calculated exclusively for responses to the stereoscopic images in congruent stereo mode, and indicates the number of stimuli in the original stimulus set to which the neuron responded, in such a way that this index was 1 when the neuron responded to only one of the stimuli, and zero if it responded equally to them all. We performed Multidimensional Scaling (MDS) analysis in Statistica (Statsoft) on the correlations between the images based on the responses of our population of AIP neurons in the Search Test. Visual inspection of the screen plot (stress as a function of the number of dimensions) allowed to determine the optimal number of dimensions in the MDS solution. For comparison, we also performed the MDS analysis on the area-equalized images using the aspect ratio (largest diameter divided by the smallest diameter) as estimates of the interstimulus distances.

In the Disparity Test, we assessed the significance of the disparity selectivity by computing an ANOVA on the responses to the congruent stereo condition, the incongruent stereo condition and the no-stereo condition (p<0.05). Consistent with previous studies the neural selectivity was considered to arise from binocular mechanisms if the neurons showed significant response differences in the stereo conditions (ANOVA p<0.05) and the response difference in the stereo conditions was at least three times larger than the response difference in the monocular conditions [7], [17]. To compare the neuronal selectivity for object images with and without disparity, we computed a 3D and a 2D Object Selectivity Index (OSI) based on the responses to the preferred and nonpreferred images in the Disparity Test: (R_preferredstimulus_ – R_nonpreferred stimulus_)/(R_preferred stimulus_). The 3D OSI was calculated based on the responses to the preferred and nonpreferred images in the preferred stereo mode (either congruent or incongruent), whereas the 2D OSI was calculated using the responses to preferred and nonpreferred images in the absence of disparity (no-stereo mode). Response latencies in the Disparity Test were calculated using the population response to the preferred image (i.e. all trials recorded for the presentation of the preferred image combined across neurons). The population response latency was defined as the first of three consecutive time bins after the stimulus onset (bin size: 20 ms) showing a significantly increased response compared to the baseline (Student’s t-test; p_value_ ≤0.05).

## Results

### Search Test

We recorded the activity of 139 AIP neurons responding to at least one of the stimuli in the Search Test (102 neurons in monkey H, 37 neurons in monkey M). Structural MRI and high-resolution ultrasound imaging were used to verify the recording positions (see Materials and Methods) and to confirm that neurons were localized in the lateral bank of the anterior IPS (Figure 1A). No saccadic activity was present at any of the recording positions, which excludes the possibility that some cells had accidentally been recorded in the most anterior region of the Lateral Intraparietal area (LIP). The absence of saccadic activity combined with the presence of selective responses to three-dimensional stimuli presented at the fixation point confirmed the recording area as AIP [7]. All AIP neurons included in this study showed significant responses to at least one of the stereoscopic images in the Search Test (t-test post- vs. pre-stimulus onset activity; p<0.05). In addition, all neurons tested showed significant discrimination (i.e. response differences) between two or more of these images (ANOVA on the net responses; p<0.05). However, these results do not imply that most AIP neurons respond to images of objects, since we observed robust responses to our set in only a limited number of grid positions in the recording chambers (3 in monkey H and 1 in monkey M) at the more posterior part of AIP. Therefore, images of objects only evoke robust visual responses in a restricted subregion of AIP.

All 139 AIP neurons were tested in the Search Test with the congruent stimulus set, but only 91 AIP neurons were tested with both the congruent and incongruent stimuli. Therefore, we present the results of the Search Test based on the responses to the congruent stereo stimuli. However, the same analyses performed on the responses to the incongruent stereo stimuli produced qualitatively similar results (data not shown). All 139 AIP neurons in the current study were responding to at least one of the stimuli in the Search Test. Stereoscopic images of real-world objects presented during passive fixation were highly effective in driving AIP neurons: the best image in the Search Test (stimulus rank = 1) produced an average response of 25spikes/sec. Furthermore, selectivity in the Search Test was robust, since our AIP population responded to 20 out of 24 congruent stereo stimuli by more than 50% of the maximum response, and the response to the least effective stimulus averaged close to 30% of the maximum response. The tuning tended to be relatively broad: the median S_width_ obtained was 0.27 (0.29 for monkey H, 0.19 for monkey M; t-test, ns) indicating that AIP cells in our study responded on average to 75% of the stimuli in the Search Test.

**Figure 2:**
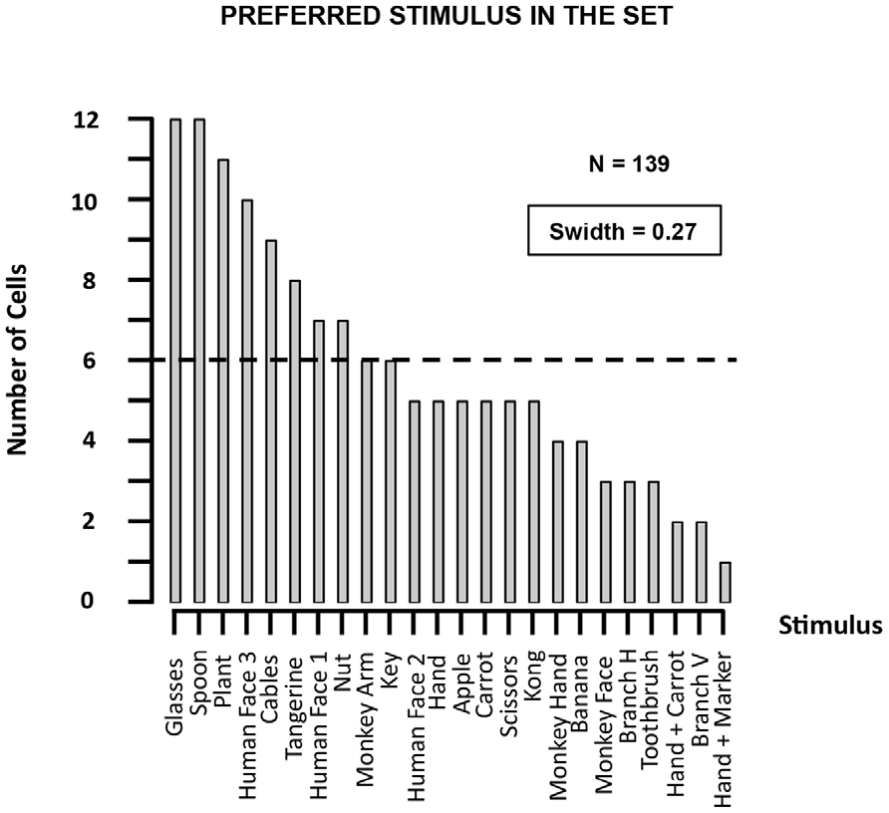
Search Test. For each stimulus in the Search Test, we plotted the number of neurons that gave the maximal response to that image. The images are ranked from the highest (left) to the lowest number of neurons preferring each particular image.

The stimuli in the original set (Search Test) differed in their effectiveness at driving AIP responses. Figure 2 shows the number of neurons preferring a given image (i.e. gave the maximum response to that image) for all stimuli in the Search Test. Some of the images of man-made objects (e.g. the spoon, the glasses), the plant, and one of the human faces were the most effective stimuli in the highest number of neurons (10–12), whereas, for example, images of a human or monkey hand were much less frequently preferred by AIP neurons (1–6 neurons). Thus these neurons respond strongly and selectively to stereoscopic images of real-world objects during passive fixation. We divided our stimulus set into two classes based on the familiarity of the object to our monkeys: familiar objects were objects which the monkeys saw regularly (e.g. apple, banana, kong, human face2), whereas unfamiliar objects (e.g. glasses, spoon, cables) were not present in their everyday life. The correlation between the familiarity and the number of neurons preferring that image was not significant (Kendall’s rank correlation coefficient r = 0.29, ns).

**Figure 3:**
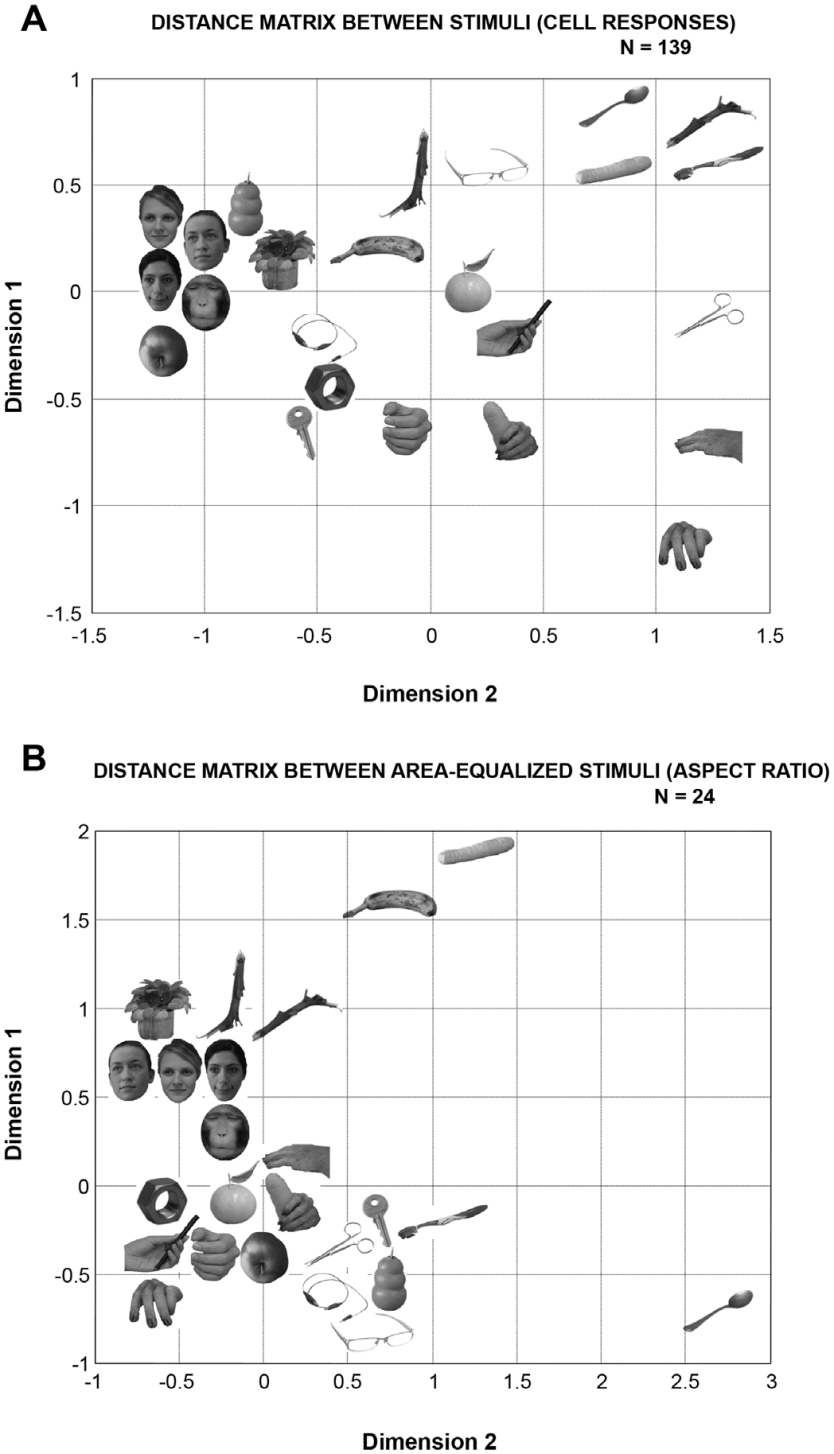
Multi-dimensional scaling (MDS) analysis. (A). Two-dimensional MDS solution using the pair-wise correlations between the images based on the net AIP responses as estimates of the inter-stimulus distances. (B). Two-dimensional MDS solution using the aspect ratio of the images (largest diameter divided by the smallest diameter).

Our stimulus set contained images of animate (faces, hands) and inanimate (both natural and man-made) objects. We wanted to determine whether the responses of this AIP population also represented these intuitive category boundaries, as neurons in the ITC do [13]. To that end, we performed MDS on the correlations between the images, based on the responses of our population of AIP neurons in the Search Test (Figure 3A). A two-dimensional solution provided a sufficiently robust fit to the data but showed no clear separation between the stimuli based on intuitive stimulus categories such as animate-inanimate. For example, the kong, the apple and the plant were located within the cluster of the faces. The most apparent stimulus feature in the neural space of AIP appeared to be elongation: while round stimuli (faces, kong, apple, plant) were clustered on the left side of the graph, elongated natural and man-made objects (spoon, carrot, horizontal branch, toothbrush) were clustered in the upper right corner, suggesting that aspect ratio may be an important determinant of the AIP responses. Furthermore, orientation may also be important, since the four elongated stimuli that clustered in the top right corner shared a similar orientation. Some images of hands (human hand, hand with marker and hand with carrot) occupied a more central position. Notice also that surface characteristics such as texture were largely ignored in our AIP population: for example, compare the different textures of the spoon, the carrot, the horizontal branch and the toothbrush, which cluster together, or the texture and shading differences in the kong and the human faces. Hence the neural space of AIP may primarily encode simple shape features such as aspect ratio and orientation. For comparison, we also performed the MDS analysis on the area-equalized images (to remove the effect of stimulus size) using the between-stimuli differences in aspect ratio (largest diameter divided by smallest diameter) as estimates of the inter-stimulus distances. (The same MDS analysis of images in the Search Test yielded qualitatively similar results, data not shown.) The two-dimensional solution of this MDS analysis coarsely resembled that obtained from the AIP responses (Figure 3B), (e.g. round stimuli on the left and elongated shapes such as the banana and the carrot on the right). Thus the representational space in AIP is most likely based on simple geometric features of the images such as aspect ratio and possibly a number of other shape elements. Although our stimulus set was relatively small, these results suggest that AIP neurons do not represent the intuitive category-membership of the stimuli, in contrast to the ITC [13]. As in our previous study [9] we divided the stimulus set into two categories based on the aspect ratio: round objects (e.g. the face stimuli, apple, tangerine) and elongated objects (e.g. the branches, carrot, banana, toothbrush). Overall, 91/139 (65.5%) AIP neurons gave the maximal response to one of the round objects compared to 48/139 (34.5%) AIP neurons that preferred one of the elongated objects. In order to verify the consistency of these responses to aspect ratio, we identified the three stimuli evoking the largest responses in each of the AIP neurons recorded. For 52 neurons (52/139∶37.4%) all three most effective stimuli belonged to the same aspect ratio class (42 neurons responded to round stimuli and 10 neurons responded to elongated stimuli). Thus a subpopulation of neurons in AIP may respond preferentially to the aspect ratio of the stimulus. A similar subpopulation of AIP cells has been previously described by Murata and colleagues [5] during fixation of three-dimensional objects presented on a turntable.

### Disparity Test

**Figure 4:**
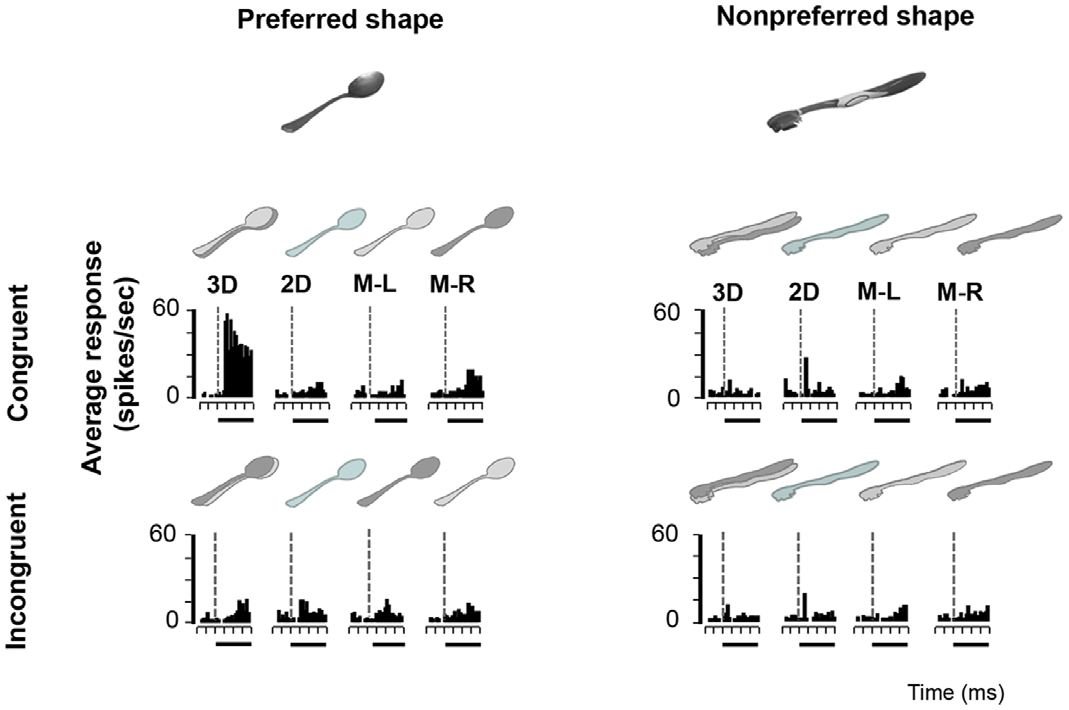
Disparity-selective example neuron. Peristimulus-time histogram (PSTH) of a neuron responding more strongly to the image of a spoon in congruent stereo mode (left top row) compared to the no-stereo mode (left second column) and incongruent stereo mode (left bottom row). No responses were measured in the monocular conditions (left columns 2–4), nor to the image of a toothbrush (right).

**Figure 5:**
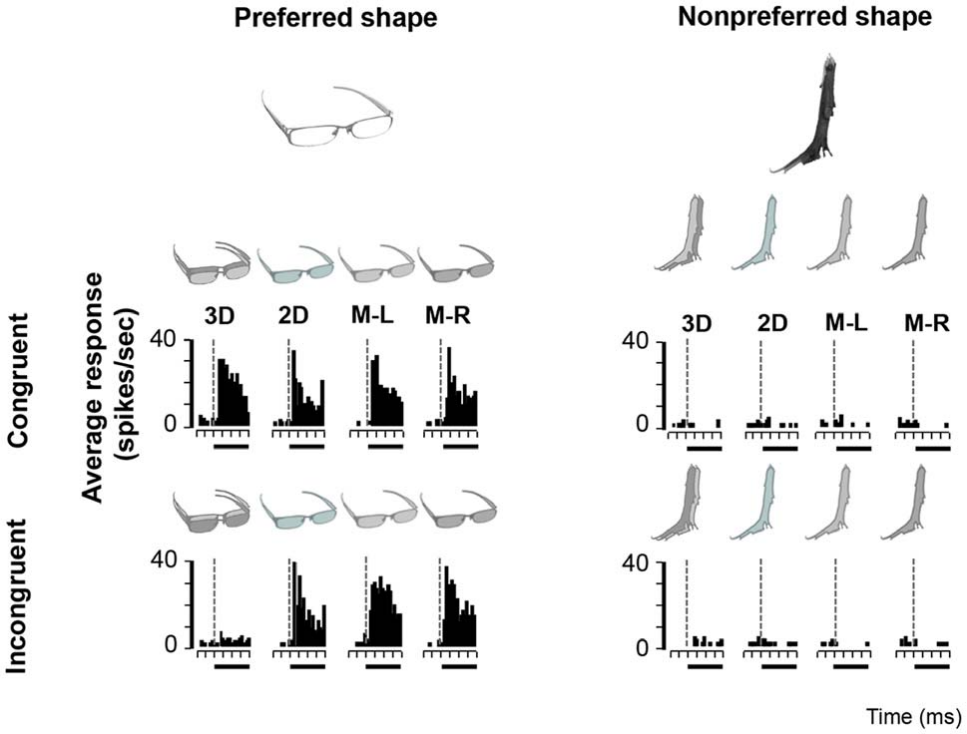
Disparity- and contour-selective example neuron. This neuron also preferred congruent over incongruent stereo stimuli (left), but remained image selective in the absence of disparity (compare the responses to the 2D image of the glasses with those of the vertical branch).

**Figure 6:**
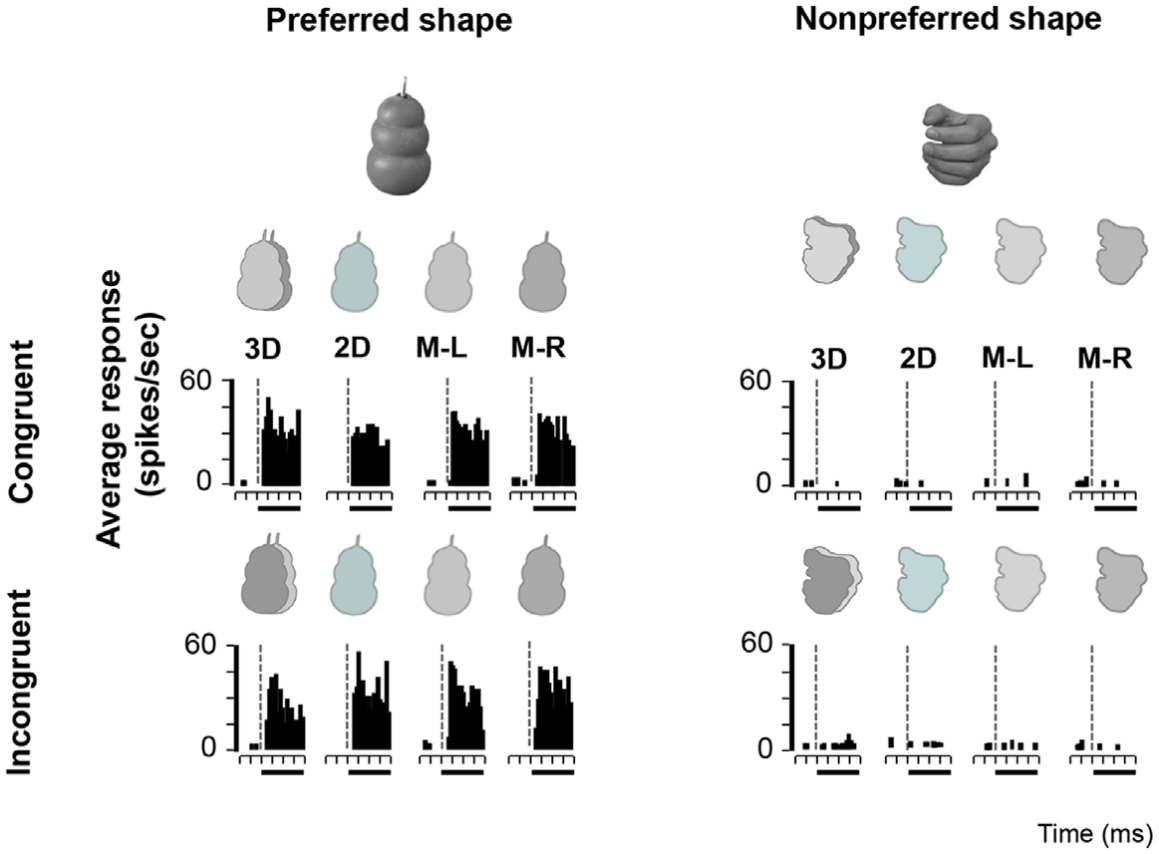
Example neuron not selective for disparity. PSTH of an example neuron that showed significant image selectivity (preferring the image of a kong over the image of a human hand) in the presence and absence of disparity.

With the Disparity Test we wanted to assess the influence of congruent, incongruent or no disparity on image selectivity in AIP neurons. All 139 AIP neurons of the Disparity Test responded significantly to at least one of the images in the Search Test. The neuron in Figure 4 responded strongly to the image of a spoon (preferred image) in congruent stereo mode, but not to the toothbrush (nonpreferred image). Removing the disparity information (no-stereo mode) almost completely eliminated the response of the neuron. Likewise, this neuron responded very weakly to the incongruent stereo mode and to monocular presentations of the preferred image. Hence for this type of AIP neuron, the presence of binocular disparity was both sufficient and necessary to evoke selective responses. Since this neuron showed significant response differences between the congruent, the no-stereo and the incongruent stimuli (assessed with ANOVA, p<0.05) which could not be accounted for by the monocular responses, this neuron was considered as disparity-selective. The second example neuron (Figure 5) also showed object-selective (preferring the image of the glasses over the image of the branch) and disparity-selective responses (preferring the congruent stereo mode), but in this case the presence of disparity was not necessary for object selectivity. In the absence of disparity information (no-stereo mode and monocular presentations), the neuron still preserved a clear selectivity for the image of the glasses over the image of the vertical branch. Therefore, for this neuron, disparity was sufficient but not necessary for producing selective responses. Although this example neuron was clearly disparity-selective, one or more other image features (e.g. 2D contour, orientation, texture) might have been also eliciting selective responses in the absence of disparity. Finally, a typical example of a disparity-nonselective neuron is shown in Figure 6. This neuron showed image selectivity responding to the image of a kong but not to a human hand. Likewise, this selectivity was independent of the stereo mode (congruent, no stereo or incongruent). For this neuron, disparity was neither sufficient nor necessary, and a different (2D) image feature or combination of features was driving its response.

Almost half of the AIP neurons recorded in the Disparity Test showed significant response differences between the congruent, no-stereo and incongruent disparity stimuli that could not be accounted for by the monocular responses, and were therefore considered disparity-selective (63/139∶45.3%; 50.5% in monkey H, 29.7% in monkey M). On average, this population of disparity-selective AIP neurons responded 39% less to the no-stereo condition compared to the preferred stereo condition. For 19 of these disparity-selective cells (19/63∶30.2%; 28.8% in monkey H, 36.4% in monkey M) disparity was both sufficient and necessary to evoke image selectivity, as in the example neuron in Figure 4. However, for the great majority of the disparity-selective AIP neurons (44/63∶69.8%; 71.2% in monkey H, 63.6% in monkey M) disparity was sufficient but not necessary for evoking selective responses. These neurons were clearly encoding both disparity information and other shape features present in the images (see example neuron in Figure 5). The remaining AIP neurons (77/139∶55.4%; 49.5% in monkey H, 70.3% in monkey M) showed a strong 2D image selectivity but these neurons were not significantly affected by disparity at all. Since our stimuli in the Disparity Test were – similar to real-world objects – unequal in size, aspect ratio, luminance and other low-level visual features, a variety of image features including the 2D contour may have contributed to image selectivity.

**Figure 7:**
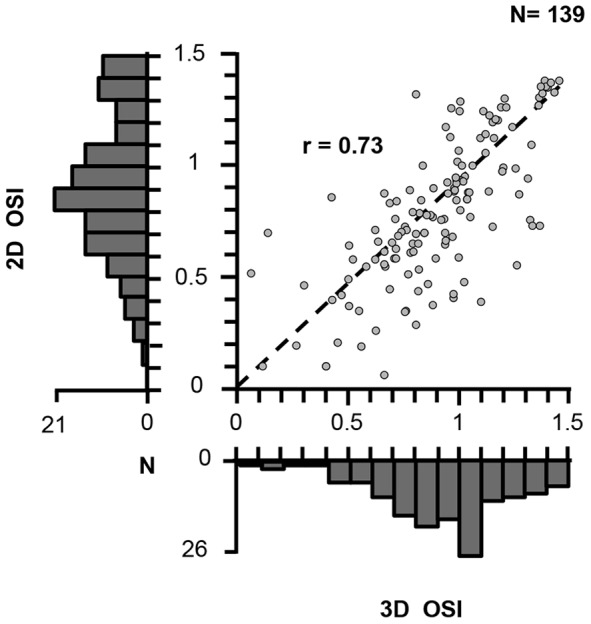
Population analysis Disparity Test. For each neuron tested we plotted the Object Selectivity Index for the images in congruent stereo mode (3D OSI, defined as (best – worst)/best) as a function of the same index in the absence of disparity (no-stereo mode, 2D OSI). The histograms show the distributions of the 3D and the 2D OSI in our population of AIP neurons.

We assessed the importance of binocular disparity on the image selectivity of AIP neurons by comparing the image selectivity (e.g. the response difference between the kong and the hand in Figure 6) in the presence (3D OSI) and absence of congruent disparity (i.e. no-stereo mode; 2D OSI, Figure 7). The correlation between the 3D OSI and the 2D OSI was high (r = 0.73, p<0.001), and only 16% of the neurons were significantly more selective in 3D (congruent disparity) than in 2D (no-stereo mode) (N = 22/139 neurons, ANOVA with *disparity* and *image* as factors, interaction, p<0.05; only 2 neurons were more selective in 2D). Thus most AIP neurons (97/139, 69.8%) responded similarly to the 3D (congruent) and 2D presentations of the stimuli (t-test, p>0.05). However, the median selectivity was higher in the presence of disparity (median 3D OSI = 0.97; median 2D OSI = 0.88), and a subpopulation of AIP neurons (38/139, 27.3%) responded significantly more strongly to 3D images compared to 2D images – a very small fraction of neurons (3%) showed the opposite preference –. These results clearly demonstrate that although many AIP neurons are sensitive to binocular disparities, the presence of disparity in images of real-world objects is rarely necessary to elicit selective responses.

**Figure 8:**
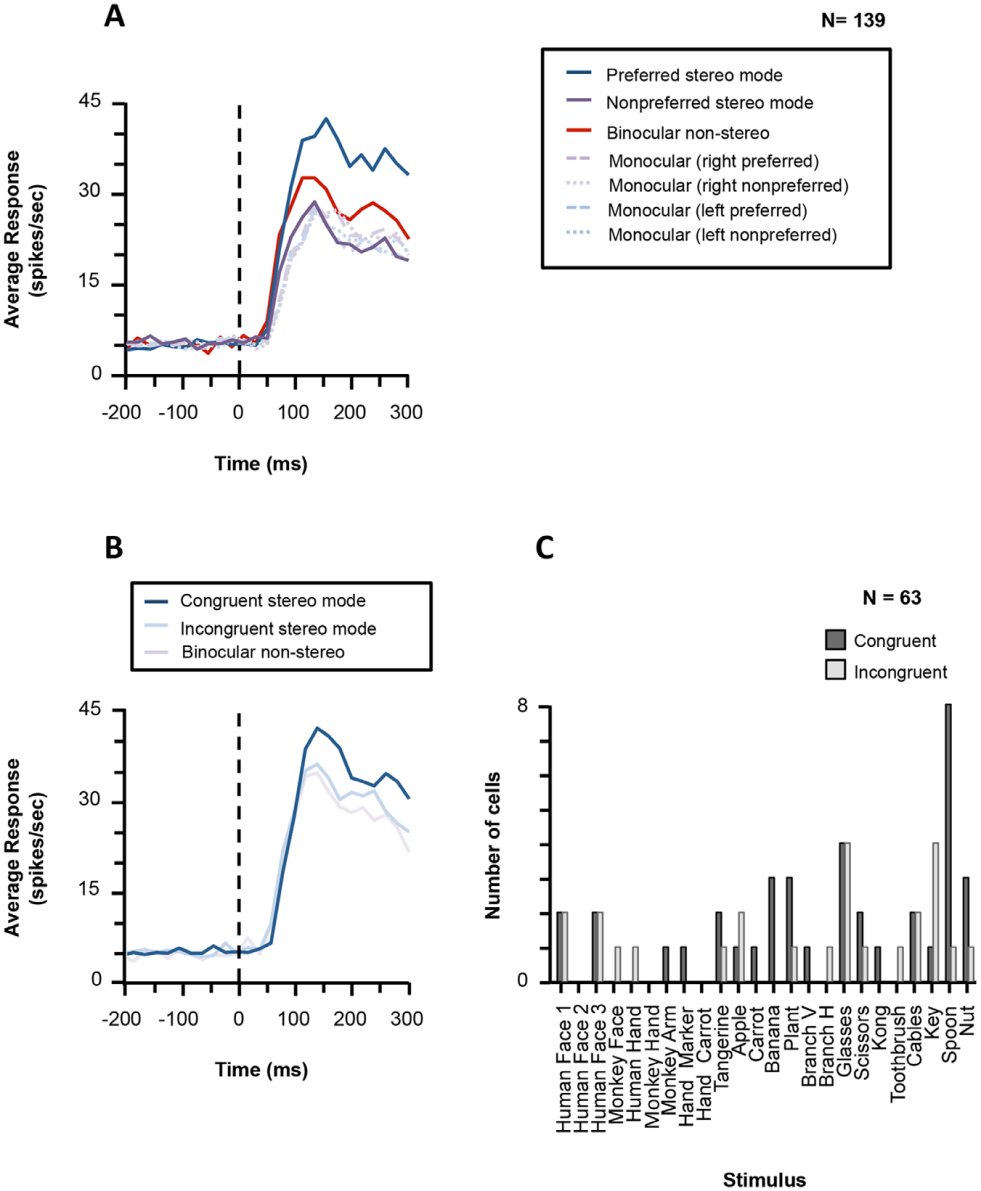
Average responses in the Disparity Test. (A). Plot showing the average population response to the preferred (dark blue line), the nonpreferred (dark purple line) and the no-stereo mode. For comparison, we also plotted the average monocular responses for each of these conditions (dashed lines). Zero indicates the time of stimulus onset. (B). The average population response is plotted for the congruent stereo mode (dark blue line), the incongruent stereo mode (light blue) and the no-stereo mode (light purple line) for all disparity-selective neurons (N = 63). (C). Histogram showing the number of neurons that preferred congruent (dark grey bars) and incongruent stereo stimuli (light grey bars) for each of the images in the Search Test.

Figure 8A shows the population response to the preferred, the nonpreferred and the no-stereo mode for all cells recorded in the Disparity Test. As expected, the average response to the non-stereo mode was intermediate between the response to the preferred and the nonpreferred stereo mode. In agreement with previous findings [7], [8], the latency of the neural selectivity for the preferred over the nonpreferred stereo mode in this study was remarkably short, occurring 60–80 ms after the stimulus onset (t-test on the population response to the preferred and nonpreferred stereo mode, first of three consecutive bins for which p<0.05).

We tested 29 disparity-selective AIP neurons with the preferred image in congruent and incongruent stereo modes presented at 3 positions in depth (0.5 deg near, 0.5 deg far and at the fixation plane). Most of these neurons (16/29, 55.2%) retained their preferences across positions indicating higher-order disparity selectivity, but the proportion of neurons that did not show invariance for position in depth (i.e. zero-order disparity cells) was larger than in previous studies [7], [8]. Thus the disparity selectivity we observed consisted of both zero-order and higher-order disparity selectivity.

We did not formally test the selectivity of AIP neurons for static monocular depth cues such as texture gradients, shading and perspective. However because all our stimuli contained monocular depth information (Figure 1B), we could assess whether AIP neurons were affected by the conflict between the monocular depth cues and the disparity information in our incongruent stereo stimuli. Despite the relatively weak influence of binocular disparity in most AIP neurons, disparity-selective AIP cells showed a moderate but significant preference for congruent over incongruent disparities (number of neurons preferring congruent disparity_ = _42; number of neurons preferring incongruent disparity_ = _21, z-test; p<0.05). As expected, the average population response to congruent stereo stimuli was stronger than that to incongruent stereo stimuli (t-test on the average activity 100–300 ms after stimulus onset; p<0.05, Figure 8B). Images of objects lacking disparity (no-stereo mode) also represent a conflict between disparity information (which signals a flat surface) and the monocular depth cues. These no-stereo stimuli also evoked less activity than the congruent stereo stimuli (t-test; p<0.05) but slightly more than the incongruent stereo stimuli (t-test, ns). The preference of AIP neurons for congruent stereo stimuli was largely confined to a small number of stimuli in our Search Test: the images of the spoon, the banana, the plant and the nut were at least four times more frequently preferred in congruent stereo mode than in incongruent stereo mode (Figure 8C) whereas for other object images there was no (e.g. the glasses) or the opposite (the key) preference of congruent over incongruent stereo mode. These results indicate that AIP neurons were indeed sensitive to depth information derived from monocular depth cues in the images: stimuli in which monocular and binocular depth cues were conflicting evoked a lower average response compared to stimuli where these two sources of depth information were congruent.

## Discussion

We investigated the role of depth information, derived from binocular disparity and static monocular depth cues, on neural selectivity for images of objects in AIP. Most AIP neurons did not require the presence of binocular disparity in the images. However, stimuli in which disparity information was congruent with the depth information in the monocular depth cues, elicited stronger responses, indicating an influence arising from monocular depth cues. This indicates that, in the representational space of AIP, object images are represented on the basis of simple shape features but not on intuitive category membership.

In contrast to previous studies using random-dot stereograms [7], [8], we tested AIP neurons with realistic stereoscopic images of real-world objects (both natural and man-made). These images were highly similar to real-world objects but had the advantage that we could manipulate the disparity content (congruent, incongruent or no-stereo mode) without changing any other image features, which allowed us to estimate the contribution of binocular disparity information to the object responses in AIP. Because we did not control the disparity content in our stimuli, interchanging the monocular images of the congruent stereo mode between the eyes may produce unexpected effects (e.g. unpaired image regions when the disparity gradients are steep). Therefore, we also tested our stimuli in no-stereo mode, in which two identical images were presented to the two eyes and no mismatch between the eyes could occur, and assessed the importance of binocular disparity by comparing the responses in the preferred stereo condition (either congruent or incongruent depending on the preference of the cell) with the responses in the no-stereo condition. The comparison between the preferred stereo and the no-stereo mode represents the most straightforward test of the importance of binocular disparity in images of real-world objects containing a rich variety of monocular depth cues.

On the other hand, the images in our stimulus set differed with regard to many features, including 2D contour, size, texture and orientation, similar to real-world objects. Because the image selectivity we observed could originate from a variety of image features, we cannot infer which of these may have been driving the selectivity. Removing disparity had a surprisingly weak influence on the image selectivity of AIP neurons, since less than one-quarter of the neurons responded significantly more strongly to their preferred image in the presence of disparity compared to the same image lacking disparity, and the average image selectivity in the presence of disparity was only marginally higher than that in the absence of disparity. These results appear to conflict with the proposed role of binocular disparity in visually-guided grasping [10] and the strong selectivity for disparity-defined 3D shapes that has been reported in previous studies [7], [8]. Note, however, that the role of binocular disparity in visually-guided grasping is reduced when grasping a small, overtrained set of objects [18]. Furthermore, the stimuli in previous studies [7], [8] were more extreme cases (random-dot stereograms with opposite disparity gradients) for which disparity was the only source of depth information. Our results demonstrate that, in natural images, binocular disparity is merely one of the many image features that can influence the responses of AIP neurons. We have to acknowledge the possibility that imperfect alignment of the two monocular images may have weakened the disparity content in some of our stimuli, and consequently we may have underestimated the influence of binocular disparity on the responses of AIP neurons. However, human observers clearly perceived at least the most effective stimuli in our set (e.g. glasses, spoon, plant, nut; congruent stereo mode) in depth.

A variety of static monocular (‘pictorial’) depth cues also carried depth information in our stimuli. Testing neural selectivity for monocular depth cues is not trivial because neuronal selectivity for particular patterns (texture, shading) can be unrelated to the 3D information they carry. As a first attempt to investigate the role of monocular depth cues in AIP, we compared neural selectivities for images in which the disparity content was either congruent or incongruent with the monocular depth cues. The moderate but significant preference we observed for stimuli in which the disparity information is congruent with the monocular depth information suggests that monocular depth cues influenced the responses of AIP neurons. Most of our stimuli were convex, and the well-known convexity bias – both at the perceptual [19] and at the neuronal level [20] – may have influenced our results to some extent. However, it is noteworthy that the image for which the effect of disparity congruence was the strongest (the spoon) was tilted in depth and was therefore a mainly first-order disparity stimulus without clear convex regions, whereas other stimuli such as the faces (convex) were no more frequently preferred in congruent versus incongruent stereo mode. Therefore, it is unlikely that the convexity bias can account for the observed preference of congruent disparity stimuli.

The modest preference of AIP neurons for congruent stereo stimuli combined with our previous observation that the large majority of AIP neurons were image selective after the removal of all texture and shading information [9], suggests that monocular depth cues are only weakly represented in AIP. These results are consistent with the monkey fMRI study of Nelissen and colleagues [21], which reported weak activations for 3D shape from texture and virtually no activations for 3D shape from shading in area AIP, in contrast to the extensive activations in the IPS evoked by curved surfaces defined by binocular disparity [22]. Furthermore, our results in AIP differ markedly from the results obtained in area CIP (Caudal Intraparietal area) [23], [24], located in the caudal IPS. Surface-orientation selective neurons in CIP exhibit strong convergence of 3D information derived from disparity, perspective and texture information. Some CIP neurons may also be selective for 3D shape [25], [26]. However it is unknown whether CIP neurons also respond to images of real-world objects.

We analyzed the results of the Search Test to investigate how a population of AIP neurons represents a set of images of real-world objects. As a population, neurons in the ITC represent the distances between object images based on intuitive object categories, most notably the animate-inanimate distinction [13]. In AIP, one of the end-stage areas of the dorsal stream, we observed a markedly different clustering of the images in the MDS solution, which ignored the animate-inanimate stimulus categories but appeared to be based largely on relatively simple shape features such as elongation and orientation. A possible exception might be the images of the hands, which occupied a separate position in the representational space of AIP. Interestingly, a recent study [14] using a continuous flash suppression paradigm provided psychophysical evidence that the human dorsal stream may preferentially process elongated shapes but not surface attributes or the tool category. Consistent with this observation, elongation was strongly represented in our AIP population whereas differences in surface characteristics (texture, shading) were largely ignored. Furthermore, the removal of surface attributes did not alter the priming effect reported by Sakuraba and colleagues [14], which nicely fits with our previous observation that silhouettes and outlines are sufficient for evoking selective responses in the great majority of AIP neurons [9]. Since our monkeys were not trained to use any of the tools in our stimulus set, we cannot draw any conclusions with respect to the representation of tools in AIP of the monkey. Furthermore, previous studies [4], [5] also showed that AIP neurons can exhibit strong selectivity to the orientation of graspable objects.

Finally, our results are consistent with prior works [5], [27] showing that AIP provides a more ‘visual’ representation of objects whereas the ventral premotor cortex represents objects in motor terms (i.e. determined by the grip type used to grasp the object). Rather than representing object categories, AIP may encode shape features that can be used to guide the pre-shaping of the hand during grasping – such as elongation and orientation –. Given that it is currently unknown whether AIP neurons respond to the entire object contour or to parts of this contour (and if so, how large these fragments would be), the identification of these critical shape features will be exceedingly difficult using complex object images or real-world objects. Future studies will have to determine the elementary shape features that drive AIP responses using a more manageable stimulus set.
